# The impact of minimal extrathyroidal extension in the recurrence of papillary thyroid cancer patients

**DOI:** 10.20945/2359-3997000000245

**Published:** 2020-06-05

**Authors:** Maria Fernanda Ozorio de Almeida, Júlia Soares Couto, Ana Luiza Trevizani Ticly, Vivian Cenize Guardia, Marilia Martins Silveira Marone, Nilza Maria Scalissi, Adriano Namo Cury, Carolina Ferraz, Rosália do Prado Padovani

**Affiliations:** 1 Irmandade Santa da Casa de Misericórdia de São Paulo São Paulo SP Brasil Irmandade Santa da Casa de Misericórdia de São Paulo, São Paulo, SP, Brasil; 2 Departamento de Medicina Nuclear Irmandade da Santa Casa de São Paulo SP Brasil Irmandade da Santa Casa de Misericórdia de São Paulo, São Paulo, SP, Brasil; Departamento de Medicina Nuclear da Irmandade da Santa Casa de São Paulo, SP, Brasil

**Keywords:** Differentiated thyroid cancer, minimal extrathyroidal extension, carcinoma extension, prognosis

## Abstract

**Objective:**

We aimed to evaluate the impact of minimal extrathyroidal extension (mETE) alone on the risk of recurrence of papillary thyroid carcinoma (PTC). The impact of other factors, including multifocality, age, tumor size, and stimulated thyroglobulin (sTg) values was also assessed.

**Subjects and methods:**

We retrospectively analyzed 1,108 PTC patients from a medical institution, who presented tumors ≤ 4 cm without any adverse characteristics other than mETE. Patients were classified according to their response to initial treatment 12 to 24 months after surgery as proposed by the 2015 American Thyroid Association (ATA) guideline. Statistical analysis was performed using multivariate logistic regression and receiver operating characteristic (ROC) curve.

**Results:**

In the multivariate logistic regression analysis, mETE did not have an impact on the response to initial treatment (p = 0.44), similar to multifocality, age, and tumor size. Initial Tg value was the only variable associated with a poor response (p < 0.01, odds ratio = 1.303, 95% confidence interval 1.25-1.36). The ROC analysis revealed that Tg was significant (area under curve = 0.8750); the cutoff value of sTg as a predictor of poor response was 10 ng/mL (sensitivity = 72.2%, specificity = 98.5%).

**Conclusion:**

For low-risk PTC presenting mETE as the only aggressive feature, the initial sTg value is essential to identify patients who may have a poor response after initial treatment and benefit from further treatment. Arch Endocrinol Metab. 2020;64(3):251-6

## INTRODUCTION

Differentiated thyroid cancer (DTC) is the most common type of thyroid cancer, and it represents approximately 90% of all thyroid malignancies ( 1 , 2 ); papillary thyroid carcinoma (PTC) is the most prevalent subtype of DTC. The prognosis of PTC patients is almost always favorable, and low-risk cases have an excellent prognosis with conservative treatment, with adequate surgery and TSH-suppressive thyroid hormone therapy ( 1 , 2 ). Approximately 3% of these patients present tumor recurrence during decades of follow-up, and in general, only 1%-2% die of these thyroid tumors ( 1 - 3 ). Therefore, radioactive iodine remnant ablation is not routinely recommended after thyroidectomy for all American Thyroid Association (ATA) low-risk DTC patients ( 2 ).

According to the ATA guideline ( 2 ), some pathological features are responsible for increasing the risk of persistence and recurrence of PTC, such as minimal extrathyroidal extension (mETE). mETE is characterized by tumor extension beyond the thyroid capsule to the perithyroid soft tissue or sternothyroid muscle alone ( 4 ), and it is observed in 5%-45% of all DTC patients ( 2 , 5 ).

Considering the initial risk stratification proposed by the ATA, the presence of mETE alone upstages low-risk patients to the intermediate-risk group, independently of age ( 2 , 6 , 7 ). It also suggests that the risk of recurrence related to mETE alone ranges from 3%-9% ( 6 , 7 ). Therefore, even when mETE is present without any other adverse features, more aggressive initial treatment is strongly recommended ( 2 , 8 , 9 ).

The ATA ( 2 ), British Thyroid Association ( 10 ) and European Thyroid Association ( 8 ) are in favor of radioactive iodine therapy (RIT) when mETE is present, whereas French Societies ( 11 ) of Nuclear Medicine and Endocrinology only recommend RIT for tumors that exhibit mETE and are larger than 1 cm ( 11 ). In addition, some studies have shown that for patients with tumors exhibiting mETE, other factors such as tumor size ( 12 - 14 ) and the value of postoperative thyroglobulin (Tg) ( 12 ) should also be considered before making a decision regarding more aggressive treatment.

Although the 8^th^ edition of the American Joint Committee on Cancer/TNM staging system has minimized the impact of minimal extrathyroidal extension (mETE), down staging it from T3 to T1 classification [compared to the 7^th^ edition ( 3 )] on the risk of death of papillary thyroid carcinoma (PTC) ( 15 ), some researchers have reported that mETE itself is related to a poor outcome ( 16 - 18 ), and major organizations ( 2 , 19 , 9 ) endorse a more aggressive management when mETE is present. So, the real role of this feature as an isolated risk factor for recurrent or persistent disease has still been questioned.

Therefore, this study aimed to assess the clinical impact of mETE as a predictor of a worse response to initial treatment in PTC tumors. As a second endpoint, we evaluated the impact of other factors such as multifocality, tumor size, and initial stimulated Tg (sTg) value on the response to initial treatment.

## SUBJECTS AND METHODS

We included PTC patients seen at the Nuclear Medicine Division at Santa Casa of São Paulo in this retrospective study. The study was approved by the local research ethics committee. The requirement for informed consent was waived, since there are no interventions on patients and most individuals no longer visit the institution. Low-risk patients and intermediate risk patients with tumors ≤ 4 cm were included. Tumors with others aggressive features as vascular invasion and aggressive histology and patients with clinically relevant lymph node metastases (cN1) ( 20 , 21 ) and positive antithyroglobulin antibodies were excluded. From the total database, 1,049 patients fulfilled the criteria to be included in the low-risk group and 59 patients in the mETE group. We included the low-risk group to evaluate the difference in risk and prognosis between the mETE group (intermediate-risk patients that receive this classification just because tumors present mETE) and the lowest risk patients corresponding the low risk stage. All patients underwent total thyroidectomy and hormone therapy with levothyroxine after surgery. No patients underwent RIT as an additional treatment. Elective lymph node dissection of the central compartment was not performed in any patient. All patients were reevaluated and reclassified 12 to 24 months after initial treatments according to the continuous risk stratification proposed by the 2015 ATA guideline ( 2 , 5 ). Excellent and indeterminate responses were considered to indicate a good response, and incomplete response included patients with biochemical and structural disease. To perform this evaluation, we used non-stimulated Tg (non-sTg), stimulated Tg values (sTg) and whole-body scan (WBS) image mainly, and other images exams as ultrasound and computed tomography when available. Since we collected data from a nuclear medicine department, we did not have complementary images from all the patients.

Serum Tg levels were assessed at least three months after total thyroidectomy with thyroid-stimulating hormone > 30 uUI/mL using the immulite Tg assay (Roche Diagnosis™, Mannheim, Germany). This is a sensitive two-site chemiluminescent immunoassay and the lower limit of detection was 0.2 ng/mL. We also analyzed the impact of some tumors characteristics as multifocality, tumor size and sTg in the response to initial treatment.

Statistical analysis was performed using multivariate logistic regression and receiver operating characteristic curve (ROC curve).

## RESULTS

Table 1 lists the clinical features of the 1,108 PTC patients included in our study. Of them, 1,049 were low-risk patients, and 59 indeterminate risk patients that had tumors ≤ 4 cm without any aggressive feature other than mETE. Women corresponded to 90.8% of the group, 75.2% of the patients were below 55 years of age at diagnosis. Regarding tumor size, the majority (61.9%) of tumors was < 1 cm, 24.1% were between 1.0 and 2.0 cm, and 14.0% were between 2.1 and 4.0 cm. The sTg measurement was available for 1,002 patients. Using the continuous risk stratification proposed by the ATA 2015 guideline ( 2 ), patients were reevaluated according to the response after 12 to 24 months from the initial treatment.

**Table 1:** Characteristics of patients with papillary thyroid carcinoma

Patient characteristics	Nº	%
Number: 1,108 patients		
Age at diagnosis		
<55 years	883	75.2
≥55 years	275	24.8
Size		
<1 cm	686	61.9
1-2 cm	267	24.1
2-4 cm	155	14.0
Sex		
Female	1,006	90.8
Male	102	9.2
Extrathyroidal extension		
No	1,049	94.7
Yes	59	5.3
Postoperative sTg values (ng/dL)		
<1	412	37.2
1-10	451	40.7
>10	139	12.5
Not stated	106	9.6
Response to initial treatment		
Excellent	29	2.6
Biochemical incomplete	152	13.7
Structural incomplete	35	3.2
Indeterminate	892	80.5

sTg: stimulated thyroglobulin; RIT: radioactive iodine therapy.

Patients with indeterminate response had non-sTg values that were detectable but < 1 ng/mL and sTg values between 1 and 10 ng/mL with no suspicious image.

We observed that 2.6% showed excellent response, 80.5% indeterminate response, 13.7% incomplete biochemistry and 3.2% incomplete structural response.

**Figure 1:**
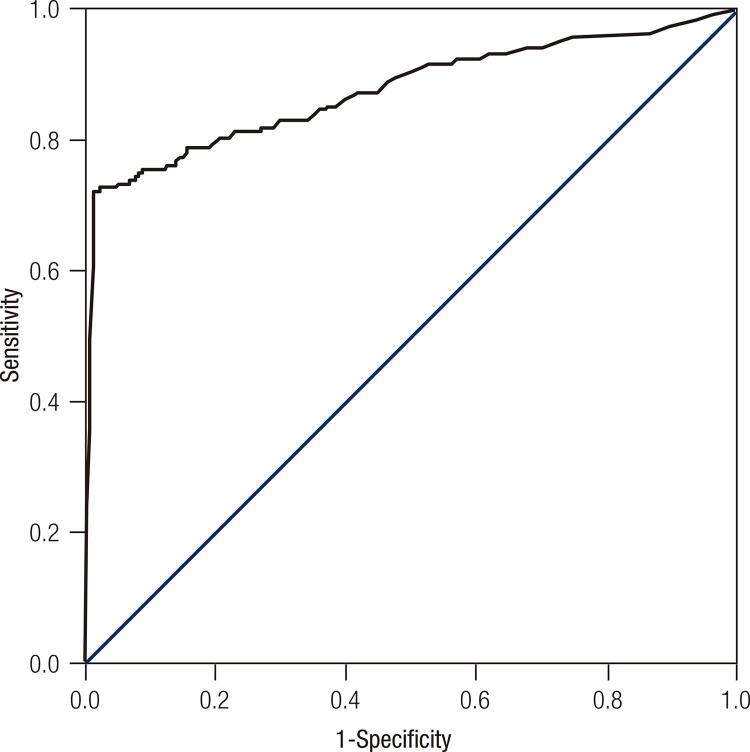
Receiver operating characteristic curve of stimulated thyroglobulin value.

In the multivariate logistic regression analysis, mETE did not have an impact on the response to initial treatment (p = 0.44), similar to multifocality (p = 0.809), age (p = 0.295), and tumor size (p = 0.385). Initial Tg value was the only variable associated with a poor response (p < 0.01, odds ratio = 1.303, 95% confidence interval 1.252-1.36) ( Table 2 ). The ROC curve analysis revealed that sTg was significant (area under the curve = 0.8750), and the cutoff value of sTg as a predictor of poor response was 10 ng/mL (sensitivity = 72.2% and specificity = 98.5%; Figure 1 ).

**Table 2:** Results of multivariate logistic regression analysis

Multivariate logistic regression analysis	

Variables	p
Age	0.295
Tumor size	0.385
Multifocality	0.809
mETE	0.444
Initial sTg	0.000

mETE: minimal extrathyroidal invasion; sTg: stimulated thyroglobulin.

## DISCUSSION

Our study evaluated the impact of some tumor characteristics as the presence of mETE, multifocality, tumor size, and initial sTg value in the response to the initial treatment of papillary thyroid cancer patients treated with surgery and not submited to RIT. sTg was the only variable that correlated with a worse response to the initial treatment excluding the impact of mETE and suggesting that the presence of this feature alone does not necessarily increase the risk of recurrence in DTC patients.

The recognition of mETE as an isolated risk factor of poor prognosis was questioned for the first time by Ito and cols. at Kuma Hospital ( 22 ). They demonstrated the lack of prognostic value of mETE alone and suggested that upgrading the category may not be appropriate ( 22 ). Following the same reasoning, the latest American Joint Committee on Cancer edition released in 2017 changed the stratification criteria and no longer considers mETE alone as an isolated risk factor for mortality ( 15 ). However, the impact of mETE on the recurrence risk remains controversial, and according to the 2015 ATA guideline ( 2 ), it should still be considered as an isolated risk factor.

Some studies have shown that mETE without concomitant gross extrathyroidal extension did not increase the rate of recurrence, similar to microscopic intrathyroidal tumors ( 23 - 27 ). However, unlike the previously cited studies, some researchers have reported contradictory results regarding the impact of mETE. A recent meta-analysis found that the presence of mETE is a factor for recurrence and decreased disease-free survival ( 16 ), supporting the indication of more aggressive management for these patients. However, the quality of some studies included in this systematic review was not much satisfactory, according to the Newcastle-Ottawa Scale score. In contrast, our results showed that mETE alone was not associated with a worse response to initial treatment suggesting that the presence of this feature alone does not necessarily increase the risk of recurrence in DTC patients.

A highlight of this study is that we analyzed a subgroup of patients with mETE and no other known aggressive feature. Furthermore, all patients analyzed, independently of sTg value, did not undergo additional treatment after surgery. This is especially important when we consider the controversy of RIT necessity in patients that presented positive sTg after surgery. The 2015 ATA guideline states that RIT treatment is generally favored for patients with T3 tumors demonstrating mETE, considered as “ATA low-to-intermediate risk” ( 2 ). Chow and cols. ( 28 ) described a cohort of 352 patients with mETE and demonstrated that RIT provided a good local control rate. In contrast, a systematic review by Lamartina and cols. ( 29 ) concluded that mETE alone is not a sufficient justification for RIT because no difference was found in the rates of disease recurrence between patients with and without RIT. We demonstrated although mETE was not considered as an isolated risk factor for a worst prognosis, patients with tumors presenting mETE and sTg < 10 ng/mL had a significant chance to respond better to initial treatment even without undergoing RIT. Moreover, patients whose tumors presented mETE and who had initial sTg > 10 ng/mL had a worst response and so, are likely to benefit from a more aggressive approach such as RIT since this group has a higher rate of incomplete response.

Concerning to the tumor size, many studies have evaluated the role of this characteristic, (specially in microcarcinomas), on the prognosis of PTC tumors ( 12 - 14 , 27 , 30 , 31 ); however, few studies found an association between tumor size and the presence of mETE ( 12 - 14 ). Buffet and cols. demonstrated that microcarcinomas presenting with mETE at diagnosis may benefit from RIT ( 30 ), and Rosario and cols. showed that mETE is related to a poor prognosis only if the tumor size is above 1.5 cm ( 12 ). We did not find any correlation between tumor size and a worst prognosis in the mETE group.

Zhi and cols. ( 14 ) showed that, in univariate and multivariate analysis, tumor size and also male gender and multifocality was significantly associated with lymph node metastasis and higher recurrence risk deserving cautious selection in surgery extent. Considering that our population has low risk features for prognosis, we could justify why we did not find any correlation between multifocality and tumor size with worst response to initial treatment, probably, in the higher risk group this data could not be extrapolated.

This study has some limitations. First, this was a retrospective analysis using data of patients who were not followed at the same center. Second, the number of patients with tumors presenting mETE was small. This is because the majority of patients with tumors presenting mETE are directed to our service to undergo RIT. Finally, an important observation is that the vast majority of patients have an indeterminate response and were not treated with radioiodine, more follow-up time and the thyroglobulin trend observation would allow stronger conclusions regarding the prognosis of this subgroup of patients. But, unfortunately, considering the data were collected from a nuclear medicine department, this information was not available for many patients.

In conclusion, this study showed that mETE alone is not associated with a worse response to initial treatment; therefore, it should not be considered as an independent risk factor for recurrent disease during the management of DTC patients. Moreover, the presence of mETE should not independently influence the decision to perform RIT. Furthermore, initial sTg after surgery should be considered in patients with tumors presenting mETE as the only aggressive feature, and additional treatment should be recommended when sTg > 10 ng/mL. If sTg is < 10 ng/mL, patients should benefit from follow-up without the need for a more aggressive approach. Our findings support the importance of individualized management of PTC patients.
